# 2024 EP Fellows Summit: Letter from the Program Directors

**DOI:** 10.19102/icrm.2025.16036

**Published:** 2025-03-15

**Authors:** William Sauer, Wendy Tzou


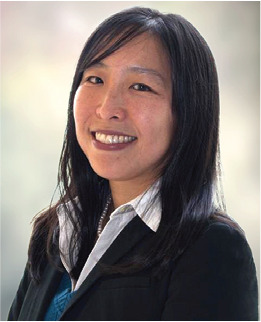



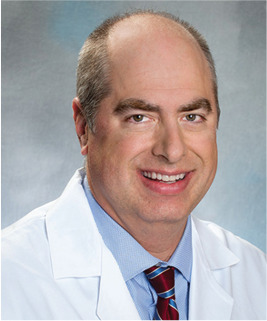


Dear readers,

As the Directors of the Electrophysiology Fellows Summit and the Arrhythmia Scholars Program, we are proud to introduce the published case reports of the three finalists who were selected to present their unique cases during the 2024 Case Competition.

As in previous years, we received numerous outstanding case reports this past year to judge. After a thorough review of the exceptional case entries submitted by fellows and residents from around the globe, the program committee nominated three finalists, below, to present their work and answer questions from the panel of judges. The winner was announced at the sessions’ conclusion following the panel deliberation and received a trophy along with a cash prize.

First, Dr. Sang Lee, a fellow at the New York Presbyterian Queens Hospital, in Flushing, NY, presented a case highlighting the challenges faced when a non-pulmonary vein source of atrial fibrillation is masked by general anesthesia. In their case report, Dr. Lee and colleagues describe their approach to this difficult situation and the eventual success with controlling atrial fibrillation in their patient.

Dr. Maxwell Coll, a fellow at Brigham and Women’s Hospital in Boston, MA, then described an unusual case of syncope due to right coronary artery vasospasm and atrioventricular block. In this case, there was clear correlation between angiographic evidence of coronary vasospasm and subsequent atrioventricular block. The case goes on to describe treatment strategies and clinical outcomes for this rare presentation.

Finally, Dr. James Mannion, a fellow at Beacon Hospital in Dublin, Ireland, presented a case on ventricular tachycardia successfully treated with pulsed-field ablation applied to tissue that was immediately heated with radiofrequency energy. Dr. Mannion hypothesized that this “stacked” energy application led to a deeper and more durable lesion compared to what would be expected using each individual energy source alone.

Congratulations to Dr. Mannion on his winning case and to Drs. Lee and Coll as the case competition finalists for their unique and interesting case presentations.

We look forward to your attendance at the 2025 EP Fellows Summit scheduled for November 7–9, 2025, at the Hyatt Regency Reston. As a hybrid conference, attendees will have the choice of attending the Summit virtually or as a traditional in-person event near Washington, DC, with the chance to participate in hands-on training sessions and interpersonal engagement. For those who are unable to attend the Summit, virtual attendance and engagement will be made possible from the convenience of your computer or mobile device through the Summit’s innovative livestreaming broadcast platforms. Detailed information will be made available at www.epfellowssummit.com.

Sincerely,

William Sauer, md

Brigham and Women’s Hospital

Harvard Medical School

Boston, MA, USA

and

Wendy Tzou, md

University of Colorado Anschutz Medical Campus

Aurora, CO, USA

